# Option B plus antiretroviral therapy adherence and associated factors among HIV positive pregnant women in Southern Ethiopia

**DOI:** 10.1186/s12884-019-2228-4

**Published:** 2019-02-28

**Authors:** Dawit Jember Tesfaye, Desalegn Tsegaw Hibistu, Teshome Abuka Abebo, Feleke Tafesse Asfaw, Kaleegziabher Lukas, Tariku Laelago, Ermias Abera Turuse, Henok Gebreyohaness Kebede, Abraham Abate Altaye, Fanuel Belayneh Bekele

**Affiliations:** 1 Department of Epidemiology, School of Public Health, College of Medicine and Health Sciences, Hawassa University, Hawassa, Ethiopia; 2 Department of Reproductive Health, School of Public Health, College of Medicine and Health Sciences, Hawassa University, Hawassa, Ethiopia; 3 Department of Health Service Management, School of Public Health, College of Medicine and Health Sciences, Hawassa University, Hawassa, Ethiopia; 4 College of Medicine and Health Sciences, Wachemo University, Hossana, Ethiopia; 5 Hossana College of Health Sciences, Hossana, Ethiopia; 6 Yale Global Leadership Institute, Yale School of Public health, YGE-GHLI New Heaven, Ethiopia; 7 Department of Midwifery, Hawassa College of Health Sciences, Hawassa, Ethiopia

**Keywords:** Antiretroviral therapy, Adherence, Option B plus, Pregnant women

## Abstract

**Background:**

Adherence to Option B plus Antiretroviral Therapy plays a vital role in preventing mother to child transmission of Human Immunodeficiency Virus and development of drug resistance. This study was aimed to assess adherence to option B plus ART and associated factors among HIV positive pregnant women at public Hospitals in Southern Ethiopia.

**Methods:**

Facility based cross sectional study was conducted on HIV positive pregnant mothers attending public health facilities’ antenatal care unit. Systematic random sampling technique was employed to select 290 HIV positive pregnant women enrolled in the Option B plus program. Data were collected by using structured questionnaire. Bivariate and multivariable logistic regression analysis were used to identify factors associated with option B plus ART adherence. *P*-value less than 0.05 was considered as cut of point to declare statistical significance.

**Results:**

The overall adherence to option B plus ART among HIV positive pregnant women was 236 (81.4%). Three in twenty, (14.8%) participants were none adherent to Option B plus ART due to difficulty in adopting time schedule and forgetting to take medication. During first trimester of pregnancy, 16 (5.5%) were stopped taking ART medication due to side effects. Pregnant women who started ART at the time of HIV diagnosis [AOR = 1.99, 95% CI: (1.02, 3.95)], and who had five or more antenatal care visits [AOR = 4.10, 95% CI (1.65, 10.02)] were more likely to adhere to option B plus ART. Women who should travel 30–60 min on foot to access ART from service delivering facilities were less likely to adhere to option B plus [AOR = 0.39, 95% C I: (0.17, 0.88)].

**Conclusions:**

The overall adherence to option B plus ART was suboptimal. Measures that improve recalling ability of individuals to take ART on time, and minimize ART side effects during first trimester of pregnancy need to be given emphasis. The study finding indicates the need for reconsidering the ad-hoc focused antenatal care visit at policy and program level by increasing the number of follow up visit with proper counseling on ART adherence benefits, and improving service accessibility.

## Background

It is evidenced that Human immunodeficiency Virus (HIV) can be transmitted from mother to child during pregnancy, childbirth, or breast feeding. The risk of transmission from mother to child is 15 to 45%. However, Antiretroviral Therapy (ART) and other effective interventions for Prevention of Mother to Child Transmission (PMTCT) of HIV can reduce risk below 5% [1]. Thus, in 2013 World Health Organization (WHO) recommended Option B plus strategy to prevent mother to child HIV transmission [2]. Based on this strategy, soon after pregnant women’s HIV positive status was confirmed, a life-long triple Antiretroviral Therapy (ART) need to be offered regardless of their CD4 count and clinical stage [3].

Globally, around 1.6 million new HIV infections among children have been prevented through provision PMTCT services since 1995. Of these, 1.3 million are estimated to have been averted between 2010 and 2015 [4]. Despite this significant progress, 23% of pregnant women living with HIV did not have access to ARVs in 2015 [5]. Consequently, in 2015 Mother to Child Transmission (MTCT) of HIV accounts for 90% of new cases among children.

In Ethiopia, Option B plus strategy for PMTCT was adopted in 2013 as a national policy to prevent MTCT of HIV/AIDS [6]. Though, the magnitude of MTCT reduction is not significant. For instance, a study conducted in southern Ethiopia revealed a 4.2% infants’ HIV positivity rate. Of these infants 2.6% were born from HIV positive mother who were on PMTCT intervention [7]. So that, country’s plan to achieve zero MTCT has been facing challenges.

In adults, 95% adherence is required to prevent MTCT, reduce the development of drug resistance and risk of treatment failure [8], and promote maternal health [9–11]. Option B plus strategy is a mechanism to improve ART adherence through the availability of a limited number of regimens, the use of fixed dose combinations, limiting unnecessary regimen switching, and selective drug taking [12, 13]. However, there are also some evidences which indicted that Option B plus PMTCT adherence would not be resolved by offering lifelong ART to all pregnant women [13].

A few studies were conducted to asses Option B plus ART adherence and associated factors in Ethiopia. The finding of these studies showed adherence level of 87.1% in Tigray region [14], 87.9% in Amhara region [15], and 81.1% in Afar region [16]. Counselling on ART medication and HIV status disclosure [14], attending a referral health center, and new diagnosis of HIV infection [15] were identified as risk factors for non-adherence. Furthermore, Loss to Follow Up (LFU) was another challenge for Option B plus ART adherence [17, 18].

Ethiopia is currently implementing prevention of mother to child HIV transmission as per to the WHO recommendation through option B plus strategy. However, there is dearth of information on ART adherence among HIV positive pregnant mothers in Southern Ethiopia. In addition, the relationship between starting time of ART and adherence is controversial and not fully understood. Therefore, this study was aimed to assess option B plus ART adherence and associated factors among HIV positive pregnant women enrolled at PMTCT program in public health facilities of Southern Ethiopia.

## Methods

### Study design, setting and population

Facility based cross sectional study was conducted among HIV positive pregnant women enrolled in option B plus PMTCT program at seven public Hospitals from March to June 2017. All selected facilities are found in Southern Nation Nationalities and People Regional State (SNNPRS) of Ethiopia. Four general Hospitals (*Adare, Yirgalem, Arbaminch, and Hosanna)*; two referral Hospitals (*Wolaita Sodo, and Dilla)*; and one health center (*Worabe*) were included in the study. As of 2017, a total of 718 pregnant women were positive for HIV in SNNPRS. Of these nearly half (48.8%) were reported from the catchment area of the study facilities.

### Study size and sampling procedure

The sample size was calculated using single population proportion formula with the following assumptions. Expected Option B plus ART adherence level of 87.1% from the study conducted in Tigray region Hospitals [14], 95% confidence level, and 4% margin of error. The calculated sample size yields 270 subjects. Considering 10% non-response rate the final sample size was 297 HIV positive pregnant women. A total of six Hospitals and one health center were purposively selected by considering the large number of HIV positive pregnant women diagnosed and enrolled in the Option B plus PMTCT program at these facilities. Probability proportional to size method was used to allocate the calculated sample size to the selected health facilities (*Adare (64), Yirgalem (36), Arbaminch (40), Hosanna (43)*, *Wolaita Sodo (45), Dilla (46), and Worabe (23)).* Systematic sampling technique was used to select study subjects (*K* = 3) until the required sample size was achieved.

### Data collection and measurements

Data were collected using structured questionnaire (with both open and closed ended questions). The questionnaire was initially prepared in English, and translated to Amharic. The Amharic questionnaire was back translated to English to check for any inconsistency in the meaning of the words and concepts. The questionnaire comprised of socio-demographic characteristics such as age, ethnicity, marital status, educational, residence and occupation. Four questions were used to assess adherence. These questions were adapted from a standardized multi method ART adherence measurement tool for low resource settings [19]. It includes; 1) Do you sometimes find it difficult to remember to take your medication during current pregnancy? 2) When you feel better, do you sometimes take a break from your medication? 3) Thinking back over the past three days, have you missed any of your doses? 4) Sometimes if you feel worse when you take the medicine, do you stop taking it? Option B plus accessibility and service related question like distance, counseling service, infant feeding options, ANC follow up, HIV disclosure status, and partner support were also included in the questionnaire. Patient chart/logbook was reviewed to collect participants CD4 count and WHO clinical stage. To minimize social desirability bias and observer bias, diploma nurses working outside the study facility were recruited. Before data collection a two day training was given for seven data collectors, and two supervisors. The training focused on the study objectives, questionnaire, and process of data collection. The data collection tool was pretested before the commencement of the actual data collection to ensure that respondents understand the questions and to check the wording, logic and skip order of the questions in a sensible way to the respondents. The respondents were interviewed after they attended ANC follow up visit in a comfortable private room that was arranged in each study hospitals for the data collection purpose. The interview took 35–45 min. The supervisors evaluated the interview through periodic observation, and reviewed the filled questionnaires for completeness and consistency.

Adherence to option B plus PMTCT was measured using self-reporting method. HIV positive pregnant women who responded “No” to the four adherence questions were considered to have good adherence level. Participants considered not adherent if responded “Yes” to one question out of four. Knowledge about option B plus PMTCT was measured based on the participant’s response to six questions. Participants who scored six considered to have high knowledge. Participants considered to have medium knowledge if they scored four or five (66.6–83.3%) out of the six knowledge question. Those who scored three and less (≤50%) were considered to have low knowledge.

### Data analysis

Data were entered in to Epi Info version 3.5.1. Then, it was cleaned and analyzed using SPSS version 20. R version 3.4.0 was used to plot graph. Variables with *p*-value of < 0.20 in bivariate analysis were selected as candidate variable for multivariable logistic regression analysis. Then, candidate variables was entered using backward stepwise method. Significant associations were reported using *p*- value and odds ratios with 95% CI. *P*-value less than 0.05 was considered as cut of point to declare presence of statistical significance. Multicollinearity among all independently associated variables was assessed. Adequacy of the final model was checked using Hosmer and Lemeshow goodness of fit test (*P* = 0.976). Finally results were presented using tables and graph.

### Ethical consideration

Ethical approval was obtained from Ethical Review Committee (ERC) of regional health bureau, Southern Nation Nationalities and Peoples Regional State (SNNPR) of Ethiopia. Verbal consent was obtained from each study participants. Participants were interviewed in private room and participation was volunteer. Identification of study participants by name was avoided to assure the confidentiality of the information obtained.

## Results

### Sociodemographic characteristics

Two hundred ninety HIV positive pregnant women taking Option B plus ART were included in the study with a response rate of 97.64%. The mean (± Standard Deviation) of the participants’ age was 28.8 (± 4.61) year. Majority, 114 (39.3%) were found in the age group 25–29 years. Urban residence accounted 238 (82.1%), and 250 (86.2%) were married. Almost one among four, 72 (24.8%) were illiterate and two third, 174 (60.0%) were housewives. Nearly half, 138 (47.6%) of the participants had a monthly income of < 1000 ETB and 44 (15.2%) had no regular income (Table 1).

**Table 1:** Sociodemographic and economic characteristics of HIV positive pregnant women enrolled in option B plus PMTCT in southern Ethiopia, 2017

Characteristics	Adherence		Total	COR, 95% CI
	Adherent	Not adheren	*N* (%)	
Age				
18–24	46 (90.2)	5 (9.8)	51 (17.6)	1
25–29	91 (79.8)	23 (20.2)	114 (39.3)	0.43 (0.15, 1.20)
30–34	81 (85.3)	14 (14.7)	95 (32.8)	0.63 (0.21, 1.86)
35–41	18 (60.0)	12 (40.0)	30 (10.3)	0.16 (0.05, 0.53)
Residence				
Rural	38 (73.1)	14 (26.9)	52 (17.9)	1
Urban	198 (83.2)	40 (16.8)	238 (82.1)	1.82 (0.9, 3.67)
Marital status				
Married	200 (80.0)	50 (20.0)	250 (86.2)	1
Not married	36 (90.0)	4 (10.0)	40 (13.8)	2.25 (0.76, 6.61)
Religion				
Protestant	107 (77.0)	32 (23.0)	139 (47.9)	1
Orthodox	107 (83.6)	21 (16.4)	128 (44.1)	1.52 (0.83, 2.81)
Others	22 (95.7)	1 (4.3)	23 (7.9)	6.58 (0.85, 50.73)
Education				
Illiterate	58 (80.6)	14 (19.4)	72 (24.8)	1
Primary	81 (73.0)	30 (27.0)	111 (38.3)	0.65 (0.32, 1.34)
Secondary	56 (90.3)	6 (9.7)	62 (21.4)	2.25 (0.81, 6.27)
Secondary+	41 (91.1)	4 (8.9)	45 (15.5)	2.47 (0.76, 8.06)
Occupation				
Housewife	145 (83.3)	29 (16.7)	174 (60.0)	1
Employed	53 (84.1)	10 (15.9)	63 (21.7)	1.06 (0.48, 2.32)
Merchant	32 (74.4)	11 (25.6)	43 (14.8)	0.58 (0.26, 1.28)
Student	6 (60.0)	4 (40.0)	10 (3.4)	0.30 (0.08, 1.13)
Monthly Income				
No income	38 (86.4)	6 (13.6)	44 (15.2)	1
≤ 1000	107 (77.5)	31 (22.5)	138 (47.6)	0.54 (0.21, 1.41)
1001–2000	35 (83.3)	7 (16.7)	42 (14.5)	0.79 (0.24, 2.58)
2001–3000	44 (84.6)	8 (15.4)	52 (17.9)	0.87 (0.28, 2.73)
> 3000	12 (85.7)	2 (14.3)	14 (4.8)	0.95 (0.17, 5.33)

### Option B plus PMTCT services and clinical characteristics

All participants were on the same triple ART regimen containing tenofovir, lamivudine, efavirenz (TDF/ 3TC/EFV). Two third of women, 184 (63.4%) were traveled on foot for < 30 min to reach health facilities for option B plus PMTCT service. Nine in ten, 265 (91.4%) women reported that they received counseling about option B plus PMTCT benefits from their service provider. Regarding infant feeding option, 260 (89.7%) of clients planned to use exclusive breast feeding. More than half 165 (56.9%) of the participants had five or more antenatal visits and knew their HIV status before their pregnancy period. Most of the study participants, 237 (81.7%) disclosed HIV serostatus to their partners/families, and 226 (77.9%) reported that they received support from their partner/family. Two hundred two (69.7%) were started ART at the time of diagnosis. One in six 49 (16.9%) participants were started ART at other health facilities (Table 2). At ART initiation, about two third 189 (66.1%) were in stage I WHO clinical category, and more than half 152 (52.4%) had > 350 cells/mm^3^ CD4 count. At the time of this study, participants with current CD4 count of ≤200, 201–350, and < 350 were 10 (3.4%), 31 (10.7%) and 161 (55.5%) respectively.

**Table 2:** Option B plus PMTCT services, and clinical characteristics of HIV positive pregnant women in Southern Ethiopia, 2017

Characteristics	Adherence		Total	COR, 95% CI
	Adherent	Not adherent	*N* (%)	
Time to reach HF				
< 30	160 (87.0)	24 (13.0)	184 (63.4)	1
30–60	36 (66.7)	18 (33.3)	54 (18.6)	0.30 (0.15, 0.61)
> 60	40 (76.9)	12 (23.7)	52 (17.9)	0.50 (0.23, 1.08)
Counseling				
Yes	221 (83.4)	44 (16.6)	265 (91.4)	3.35 (1.41, 7.94)
No	15 (60.0)	10 (40.0)	25 (8.6)	1
Infant feeding option				
Exclusive/formula	217 (82.8)	45 (17.2)	262 (90.3)	2.28 (0.97, 5.37)
Mixed	19 (67.9)	9 (32.1)	28 (9.7)	1
ANC visit				
Second	37 (67.3)	18 (32.7)	55 (19.0)	1
Third	34 (82.9)	7 (17.1)	41 (14.1)	2.36 (0.88, 6.36)
Fourth	18 (62.1)	11 (37.9)	29 (10.0)	0.80 (0.31, 2.03)
Five+	147 (89.1)	18 (10.9)	165 (56.9)	3.97 (1.88, 8.38)
Knowledge of HIV status				
Known previously	129 (78.2)	36 (21.8)	165 (56.9)	1
Current pregnancy	99 (87.6)	14 (12.4)	113 (39.0)	1.97 (1.01, 3.86)
Another pregnancy	8 (66.7)	4 (33.3)	12 (4.1)	0.56 (0.16, 1.96)
Partner notification				
Yes	192 (81.0)	45 (19.0)	237 (81.7)	2.67 (0.83, 8.54)
No	8 (61.5)	5 (38.5)	13 (4.5)	1
Partner support				
Yes	185 (81.9)	41 (18.1)	226 (77.9)	2.71 (1.11, 6.61)
No	15 (62.5)	9 (37.5)	24 (8.3)	1
Started ART at diagnosis				
Yes	173 (85.6)	29 (14.4)	202 (69.7)	2.37 (1.29, 4.35)
No	63 (71.6)	25 (28.4)	88 (30.3)	1
Start ART at other facility				
Yes	32 (65.3)	17 (34.7)	49 (16.9)	0.34 (0.17, 0.68)
No	204 (84.6)	37 (15.4)	241 (83.1)	1
Knowledge on Option B+				
Low	47 (74.6)	16 (25.4)	63 (21.7)	
Moderate	93 (82.3)	20 (17.7)	113 (39.0)	1.58 (0.75, 3.33)
High	96 (84.2)	18 (15.8)	114 (39.3)	1.82 (0.85, 3.88)

### Option B plus antiretroviral adherence

The overall adherence level of the participants was 236 (81.4%) [95% CI: (76.2, 86.2)]. Forty three (14.8%) of the participants were none adhered to Option B plus ART due to difficulty in adopting time schedule and forgetting to take medication. One in seventeen, 17 (5.9%) were interrupted taking ART medication at least once. During first trimester of pregnancy 16 (5.5%) were stopped ART medication due to side effects. Eight participants (2.8%) were missed ARV doses in the last 3 days.

**Fig. 1:**
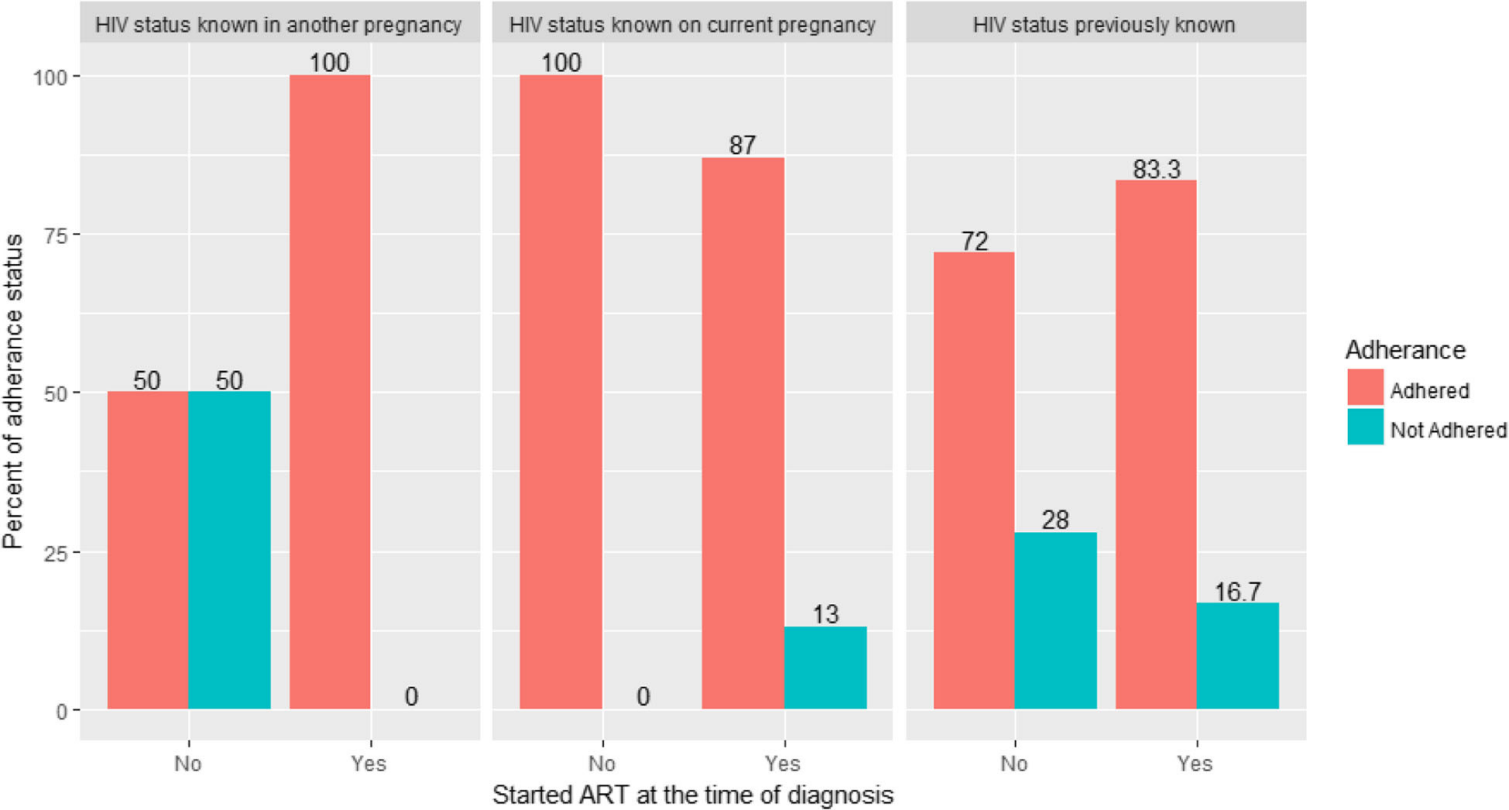
Option B plus adherence level by knowledge of HIV status during PMTCT enrolment and timing of ART initiation among HIV positive pregnant women in Southern Ethiopia, 2017. The X-axis represents status of ART initiation at HIV diagnosis; the Y-axis represents percent of adherence level; the left column shows women diagnosed for HIV at another pregnancy, the middle column shows women newly diagnosed for HIV during current pregnancy, and the right column represents women who knew their HIV positivity in non-pregnancy period

Among participants who knew their HIV status before PMTCT enrollment and started ART at the time of diagnosis, 83.3% of them had good adherence to Option B plus drugs, while 16.7% were non-adherent. Most 87.0% of mothers who were diagnosed in the current pregnancy and started ART at the time of diagnosis showed good adherence. However, in this group, 100% of mothers who were not started ART at HIV diagnosis showed good adherence level. One in twenty four, 12 (4.1%) knew their HIV serostatus during their previous pregnancies. In this group, mothers who exhibits good and poor adherence level was equal (50%) among those who were not started ART at the time of diagnosis. However, mothers who knew their HIV status during their previous pregnancies and started ART at HIV diagnosis had 100% adherence (Fig. 1).

### Factors associated with adherence to option B plus ART

The multivariable logistic regression analysis result showed that, starting time of ART, number of antenatal care visits, and time to reach health facility had significant association with option B plus ART adherence level.

HIV positive pregnant women who started ART at the time of diagnosis were two times more likely to adhere to option B plus ART than mothers who were not started ART at the time of diagnosis [AOR = 1.99, 95% CI: (1.02, 3.95)].

Those participants who had five or more ANC visits were four times more likely to adhere to option B plus ART than HIV positive pregnant women who had two antenatal visit [AOR = 4.10, 95% CI (1.65, 10.02)].

Compared to HIV positive pregnant women who traveled for less than 30 min on foot to reach health facility for Option B plus services, those participants who traveled 30–60 min were less likely to adhere to option B plus ART [AOR = 0.39, 95% C I: (0.17, 0.88)] (Table 3).

**Table 3:** Multivariable logistic regression analysis model predicting option B plus ART adherence level among HIV positive pregnant women in Southern Ethiopia, 2017

	Adherence category		COR, 95% CI	AOR, 95% CI
	Adherent	Not Adherent		
Started ART at diagnosis				
Yes	173 (85.6)	29 (14.4)	2.37 (1.29, 4.35)	1.99 (1.01, 3.95)
No	63 (71.6)	25 (28.4)	1	1
Counseling				
Yes	221 (83.4)	44 (16.6)	3.35 (1.41, 7.94)	1.72 (0.54, 5.45)
No	15 (60.0)	10 (40.0)	1	1
ANC visit				
Second	37 (67.3)	18 (32.7)	1	1
Third	34 (82.9)	7 (17.1)	2.36 (0.88, 6.36)	1.81 (0.59, 5.52)
Fourth	18 (62.1)	11 (37.9)	0.80 (0.31, 2.03)	0.96 (0.34, 2.69)
Five+	147 (89.1)	18 (10.9)	3.97 (1.88, 8.38)	4.10 (1.65, 10.02)
Partner notification				
Yes	45 (19.0)	192 (81.0)	2.67 (0.83, 8.54)	3.37 (0.94, 12.00)
No	5 (38.5)	8 (61.5)	1	1
Time to reach HF (minute)				
< 30	24 (13.0)	160 (87.0)	1	1
30–60	18 (33.3)	36 (66.7)	0.30 (0.15, 0.61)	0.39 (0.17, 0.88)
> 60	12 (23.7)	40 (76.9)	0.50 (0.23, 1.08)	1.09 (0.44, 2.66)

## Discussion

In this study, we identified the adherence level of option B plus ART and associated factors among HIV positive pregnant women. The associated factors were related to timing of ART initiation, number of antenatal care visits, and time to reach service delivering health facilities.

The overall adherence level (81.4%) was suboptimal, compared with the recommended adherence level of 95% and above to prevent vertical transmission of HIV and drug resistance [8]. The finding is similar with other studies conducted in Nigeria (80.6%) [20], Kenya (82%) [21], Zambia (82.5%) [22], and Ukraine (86%) [23].

In contrary, the adherence level in this study is higher than a 72.9% adherence rate reported in the studies conducted at Tikur Anbesa Specialized Hospital, Addis Ababa, Ethiopia [24], and 73.3% in Sobi Specialist Hospital, Ilorin, Nigeria [25]. This might be due to the difference in the study population where the other studies were conducted among People Live with HIV/AIDS (PLHIV). It is indicted that HIV positive pregnant mother demand to have HIV free child than the general PLHIV [15] might be the reason for the higher adherence to option B plus ART in this study.

However, our finding is lower than findings of different studies conducted in Ethiopia; Tigray region 87.1% [14], South Wello zone of Amhara region 87.9% [15], and Malawi 91% [26]. This might be due to the difference in the inclusion of study subjects. Unlike our study, mothers who knew their HIV positivity status before pregnancy were excluded in the Tigray study. Thus, these mothers tends to have prolonged exposure for ART that might negatively influence adherence level. The result of our study also showed that the non-adherence level (21.8%) among those who knew their HIV positivity status before they become pregnant was higher than those who knew during their current pregnancy period (12.4%). In the case of the Amhara region study [15], the reason might be difference in measurement of adherence.

Starting ART at the time of diagnosis was significantly associated with good adherence to option B plus ART. To the contrary, a study from South Wello zone of North east Ethiopia reported that HIV-positive pregnant and breastfeeding mothers who faced challenges in testing and initiating option B plus treatment at the same day of HIV diagnosis was associated with poor adherence [15]. Another study from Northeast Ethiopia also reported that women who started ART on the day they were diagnosed had a higher risk of LFU than women who started ART later [17].

In this study, the relationship of ART initiation time and adherence level was differ by knowledge of HIV status. Pregnant women who have known their HIV status during current pregnancy and who do not started ART at the time of the diagnosis had a relative good adherence level (100%) as compared to those who initiated ART at the time of the diagnosis (87%). Similarly, a study from Zambia showed women newly diagnosed as HIV positive were less likely to adhere to option B plus ART [22]. This could be due to the combined effect of ART side effects at the time of initiation and pregnancy induced physiological side effects such as; nausea and vomiting. A qualitative study from Malawi also showed initiating ART on the same day as learning their HIV status and the need to obtain HIV test at another facility to confirm their HIV status was another concerns on option B plus PMTCT [27].

Those participants who had five and above ANC visit were more likely to adhere to option B+ ART than HIV positive pregnant women who had two visits. This may be due to the fact that frequent contact with health care provider increase the opportunity to be counselled about ART adherence. The proportion of participants who received counseling about proper ART adherence and benefits during the second (17.0%) and fifth (57.7%) ANC visits in this study could also justify the difference in the opportunity for counseling.

Compared to HIV positive pregnant women who traveled for less than 30 min to reach health facility for option B plus services, those participants who traveled 30–60 min were less likely to adhere to option B plus ART. This is in line with the study conducted in Nepal [28] where those clients travelled more than 1 h to hospital were more likely to be non-adherent. A systematic review and meta-Analysis also showed distance to clinic is one of the patient-reported barriers to ART adherence [29]. This finding indicates the need for improving accessibility of Option B plus PMTCT services to all HIV positive pregnant women in the region.

## Strength and limitation

Despite using primary data and validated adherence measurement tool, interpretation of the results needs consideration of the following limitations. In this study adherence was measured using participants self-report as a result the adherence category may be subjected to recall bias and misclassification bias. However, self-reporting is the most commonly used measure of adherence in resource limited settings because it is easy to conduct in routine clinical practice. To address limitation with regard to generalizability, attempts have been done to include participants from multiple health facilities in the region.

## Conclusions

In this study, the overall adherence to option B plus ART among HIV positive pregnant women was suboptimal. Measures that improve recalling ability of individuals to take ART on time, and minimize ART side effects during first trimester of pregnancy need to be given emphasis. The study finding indicates the need for improving ART adherence through initiating ART at the time of HIV diagnosis, better service accessibility, and strengthening the current antenatal care with proper counseling on ART adherence benefits. Increasing the number of ANC follow up visit for HIV positive pregnant women have a positive effect on option B plus adherence level. The finding implies the need to reconsider the ad-hoc focused antenatal care visit at policy and program level by increasing the number of follow up visit. In addition, future investigation on how to improve ANC utilization in the local context is important to advance level of adherence.

## Abbreviations

AIDS: Acquired Immune Deficiency Syndrome
ANC: Antenatal Care
AOR: Adjusted Odd Ratio
ART: Antiretroviral Therapy
CI: Confidence Interval
COR: Crude Odd Ratio
ETB: Ethiopia birr
HIV: Human Immune Deficiency Virus
LFT: Loss to Follow Up
PMTCT: Prevention of Mother to Child Transmission
SNNPR: South Nation Nationalities and Peoples Region
SPSS: Statistical Package for Social Science
WHO: World Health Organization
